# Apigenin inhibits NLRP3 inflammasome activation in monocytes and macrophages independently of CD38

**DOI:** 10.3389/fimmu.2024.1497984

**Published:** 2025-01-07

**Authors:** Knut Husø Lauritzen, Kuan Yang, Michael Frisk, Mieke C. Louwe, Maria Belland Olsen, Mathias Ziegler, William E. Louch, Bente Halvorsen, Pål Aukrust, Arne Yndestad, Øystein Sandanger

**Affiliations:** ^1^ Research Institute of Internal Medicine, Oslo University Hospital, Rikshospitalet and University of Oslo, Oslo, Norway; ^2^ Institute of Clinical Medicine, Faculty of Medicine, University of Oslo, Oslo, Norway; ^3^ Institute for Experimental Medical Research, University of Oslo and Oslo University Hospital, Oslo, Norway; ^4^ Department of Biomedicine, University of Bergen, Bergen, Norway; ^5^ Section of Dermatology, Oslo University Hospital, Rikshospitalet, Oslo, Norway

**Keywords:** NLRP3, CD38, apigenin, inflammasome, monocyte, macrophage

## Abstract

**Introduction:**

CD38, a regulator of intracellular calcium signalling, is highly expressed in immune cells. Mice lacking CD38 are very susceptible to acute bacterial infections, implicating CD38 in innate immune responses. The effects of CD38 inhibition on NLRP3 inflammasome activation in human primary monocytes and monocyte-derived macrophages have not been investigated. Apigenin is a naturally occurring flavonoid known to inhibit CD38. However, apigenin has also been proposed to inhibit the extracellular ATP receptor P2XR7, an upstream activator of NLRP3. In this study we aimed to investigate whether apigenin attenuates NLRP3 inflammasome activation in human monocytes and monocyte-derived macrophages through CD38 inhibition.

**Methods:**

LPS-primed human monocytes and monocyte-derived macrophages were treated with apigenin, the CD38 inhibitor 78c, antagonists of CD38 second messengers (8-br-ADPR and 8-br-cADPR) or the ATP hydrolase, apyrase, prior to NLRP3 activation with ATP, monosodium urate crystals (MSU) or nigericin. IL-1β and TNF secretion and mRNA expression, as well as N-terminal gasdermin-D formation were quantified. Ca^2+^ mobilization was determined by live confocal microscopy. NLRP3 activity was also compared in WT and CD38^-/-^ mouse bone marrow-derived macrophages (BMDMs) with and without CD38 inhibitors.

**Results:**

Apigenin significantly inhibited IL-1β release from LPS-primed monocytes and macrophages activated with ATP, MSU, or nigericin. CD38 inhibition with 78c also attenuated NLRP3-dependent IL-1β release. Apigenin was a potent inhibitor of Ca^2+^ flux from the endoplasmic reticulum to the cytosol in human monocyte-derived macrophages. Apyrase attenuated IL-1β release induced by ATP or MSU, but not by nigericin. However, the NLRP3 inflammasome is not compromised in CD38^-/-^ bone marrow-derived macrophages compared to corresponding WT cells, and apigenin moderated IL-1β release in both genotypes.

**Discussion:**

Our data support that apigenin attenuates NLRP3 activation independently of CD38. Our results also suggest that MSU crystals activate NLRP3 through autocrine or paracrine ATP signalling.

## 1 Introduction

Interleukin-1 (IL-1)-related molecules and signaling pathways are crucial for our immune system. They amplify inflammatory responses against infection and injury, induce emergency myelopoiesis, and enhance antigen-driven adaptive immune responses in general and type 3 immune responses (e.g., Th17 driven responses against extracellular pathogens) in particular (1, 2). IL-1β, the “master” cytokine in the IL-1 family, is highly produced by monocytes and macrophages during acute inflammatory responses (3). Although essential for host defense, IL-1β can also contribute to the pathogenesis of several non-infectious diseases characterized by acute or persistent inflammation, including autoinflammatory periodic fever diseases, autoimmune disorders and cardiovascular disease such as atherosclerotic disorders (4–6). Thus, its secretion is tightly regulated and requires two independent events to occur (7). First, inactive pro-IL-1β is transcribed and translated in response to innate immune receptors (e.g., toll-like receptors [TLRs]) or cytokine receptors activating the transcription factor nuclear factor κB (NFκB) (signal 1). The second event is inflammasome-mediated post-translational activation. In the case of tissue damage, this is mediated by NOD-like receptor with a PYD-domain 3 (NLRP3) which can be activated by a wide range of chemically different danger signals (signal 2) such as extracellular ATP, crystals including those precipitated from extracellular urate, or pore-forming bacterial toxins such as nigericin (8). All NLRP3 activating signal pathways seem to converge at a state of reduced cytosolic potassium concentration and mitochondrial reactive oxygen species (ROS) formation (9). NLRP3 inflammasomes, consisting of NLRP3, apoptosis-associated speck-like protein containing a caspase recruitment domain (ASC) and autolytically activated caspase-1, then cleaves pro–IL-1β into active IL-1β. Caspase-1 also facilitates IL-1β release by cleaving gasdermin-D, from which multimers of the N-terminal peptide forms pores in the plasma membrane (10).

CD38 is a multifunctional enzyme with both hydrolase and cyclase activity that catalyzes the conversion of nicotinamide adenine dinucleotide (NAD) to adenosine diphosphoribose (ADPR) or to a lesser extent cyclic ADPR (cADPR) and nicotinamide (NAM) (11). Both ADPR and cADPR serve as intracellular second messengers mediating Ca^2+^ influx to the cytosol: ADPR activates the Ca^2+^ channel Transient Receptor Potential Melastatin 2 (TRPM2) expressed on the plasma membrane and on endolysosomes (12); cADPR activates ryanodine receptors (RyR) on the endoplasmic reticulum (ER) (11, 13). Remarkably, CD38 appears to be expressed both as a transmembrane ectoenzyme and a transmembrane endoenzyme, featuring enzymatic activity in both compartments (13). Furthermore, CD38 is highly expressed in immune cells including monocytes and inflammatory (M1) macrophages and is up-regulated during cell activation (14, 15). CD38 inhibition has been shown to attenuate lipopolysaccharide (LPS)-induced M1 polarization of human macrophages *in vitro* (15) and CD38 knockout mice are highly susceptible to *L. monocytogenes* and *S. pneumoniae* infection (16, 17). However, it is unclear whether CD38 is involved in NLRP3 inflammasome activation, and if so, in what way. On one hand, increased NLRP3 activity has been observed in smooth muscle cells from CD38 knockout mice (18). Recently, however, CD38 was reported to mediate NLRP3 inflammasome activation in smooth muscle cells from diabetic mice (19).

Apigenin (4′,5,7-trihydroxyflavone), a flavonoid present in vegetables and fruits, has been shown to inhibit both the NAD^+^ase activity and the ADP-ribosyl-cyclase activity of CD38 *in vitro* and *in vivo* (20, 21). Apigenin can reduce inflammation by attenuating NFκB activation (signal 1) (22–24). However, apigenin and other flavonoids have also been shown to decrease NLRP3-dependent IL-1β release from THP-1 cells (25). Flavonoids have been proposed to mediate this effect by inhibiting P2X purinoceptor 7 (P2X7R), although this remains to be shown (25).

CD38 and NLRP3 inflammasomes are two important parts of intracellular machinery regulating inflammation, but if these pathways interact and in what way is still elusive. To further elucidate the potential role of CD38 in regulation of NLRP3 inflammasome activation, we investigated whether apigenin attenuates NLRP3 inflammasome activation in human monocytes and monocyte derived macrophages through CD38 inhibition.

## 2 Materials and methods

### 2.1 Reagents

Ultrapure LPS from Escherichia coli (O111:B4), MSU and nigericin were purchased from InvivoGen (Toulouse, France). Apigenin, ATP and apyrase were bought from Sigma-Aldrich (Darmstadt, Germany). 78c was purchased from MedChemExpress (Monmouth Junction, NJ). 8-bromo-ADPR and 8-bromo-cADPR were bought from Biolog (Bremen, Germany).

### 2.2 Monocytes isolation and macrophage differentiation

Fresh buffy coats were obtained from Oslo University Hospital blood bank. Peripheral blood mononuclear cells (PBMCs) was isolated by gradient centrifugation with Lymphoprep (STEMCELL Technologies, Cambridge, UK), from which monocytes were isolated by plastic adherence method as previously described (26). The cells were washed twice with Roswell Park Memorial Institute (RPMI) 1640 (Invitrogen) to remove lymphocytes (purity of >90% monocytes) (27). Monocytes were cultured at 37˚C, 5% CO_2_ in dishes (Nunclon Delta surface; Thermo Fisher Scientific, Waltham, MA) with RPMI 1640 medium containing 25 mM HEPES and stable glutamine, 10% heat-inactivated FBS (Biowest), 5 U penicillin/ml, and 50 mg/ml streptomycin (Sigma-Aldrich) at 300,000 cells/ml. Macrophages were differentiated from monocytes by 20 ng/mL human macrophage colony-stimulating factor (M-CSF, R&D Systems, Minneapolis, MN) for 7 days and the medium was replaced on day 3 and day 6.

#### Cell culture experiments

Cells were primed with 10 ng/mL LPS for 5 hours (3.5 hours with MSU), then incubated for 30 minutes with apigenin (100 µM), 78c (10 µM), 8-bromo-ADPR (100 µM), 8-bromo-cADPR (100 µM) or apyrase (10 U/mL) before NLRP3 inflammasome activation with ATP (3 mM for 30 minutes), MSU (200 µg/mL for 2h) or nigericin (10 µM for 30min). Cell-free supernatants and protein/RNA were harvested and stored at -80°C before analyzing.

### 2.3 Ca^2+^ imaging

LPS-primed macrophages were incubated with apigenin, 8-bromo-ADPR or 8-bromo-cADPR as described above, then labeled with Fluo-4 AM (20 µM, Thermo Fisher Scientific) for 5 minutes at room temperature. LSM 7 Live confocal microscope (Zeiss) performed live cell imaging at 37˚C, 5% CO_2_. 10 random views were taken in every 10 seconds for 10 minutes. ATP (3 mM) was added after 1 minutes of recording. Signal intensity was quantified by Fiji, an image processing package based on ImageJ (28).

### 2.4 Cytokine, cytotoxicity, NAD^+^ quantification

DuoSet ELISA Kits (R&D Systems) were used to measure IL-1β and TNF release in cell-free supernatants. The supernatant was also used to determine cytotoxicity with ToxiLight BioAssay Kit (Lonza, Alpharetta, GA). Positive controls were generated by lysis of control cells with ToxiLight™ 100% lysis reagent, while volumes in non-lysed samples were corrected with the included tris acetate buffer (Lonza, Alpharetta, GA). NAD^+^ in macrophages was measured by NAD/NADH-Glo assay kit (Promega, Madison, WI). All assays were carried out according to the manufacturer’s manual.

### 2.5 mRNA quantification

RNA was isolated with the RNeasy Mini Kit (QIAGEN) and cDNA was synthesized by qScript cDNA SuperMix (Quantabio, Beverly, MA) as according to the manufactures protocol. Real-time PCR was performed with PerfeCTa SYBR Green SuperMix Low ROX (Quantabio) on 7900HT Fast Real-Time PCR System (Thermo Fisher Scientific). Primer sequences are listed in **Table 1**.

**Table 1:** PCR primer sequences.

Target	Species	Sequence (5’ 3’)	Acc. no.
IL-1β	Human	Forward: 5′-CCCTAAACAGATGAAGTGCTCCTT-3′ Reverse: 5′-GGTGGTCGGAGATTCGTAGCT-3′	NM_000576
TNF	Human	Forward: 5′-CCAGGCAGTCAGATCATCTTCTC-3′ Reverse: 5′-GGAGCTGCCCCTCAGCTT-3′	NM_000594
GAPDH	Mouse/rat/human	Forward: 5′-CCAAGGTCATCCATGACAACTT-3′ Reverse: 5′-AGGGGCCATCCACAGTCTT-3′	NM_008084

### 2.6 Immunoblot analysis

RIPA Buffer (Sigma-Aldrich) containing Halt Protease and Phosphatase Inhibitor (Thermo Fisher Scientific) was used to extract protein. Pierce BCA Protein Assay Kit (Thermo Fisher Scientific) was used to determine protein concentration. Protein samples were reduced, denatured, separated by SDS-PAGE and transferred to PVDF membrane. Radiance Plus (Azure Biosystems, Dublin, CA) was used to develop the membranes. Images were captured by Azure Imaging system model 600 (Azure Biosystems) and quantified by Image Studio Lite (version 5.2; Li-Cor, Lincoln, NE). Antibodies involved: anti–IL-1b (MAB601, R&D Systems), anti-gasdermin D (G7422, Sigma-Aldrich), anti-β-actin (A5441, Sigma-Aldrich), anti-rabbit IgG (7074, Cell Signaling Technology, Leiden, Netherlands), anti-mouse IgG (7076, Cell Signaling Technology).

### 2.7 Animal experiments

The CD38^-/-^ mouse strain (B6·129P2-CD38tm1Lnd/J) was obtained from The Jackson Laboratory and back-crossed into C57BL6/J for ten generations to obtain heterozygous founder mice (genotyping primers listed in **Supplementary Table 1**). The resulting wild type (WT) and CD38^-/-^ littermates were used as the P0 generation for further breeding. All experiments were conducted using WT and CD38^-/-^ mice from the P1 generation. Housing conditions were a 12-h light-dark cycle with ad libitum access to food and water unless otherwise stated. All mice were genotyped (**Supplementary Figure 1**).

### 2.8 Bone Marrow–Derived Macrophages

Bone marrow was isolated by flushing femurs and tibiae and culturing cells in RPMI 1640 medium (Invitrogen) containing 10% heat‐inactivated FCS, 100 U/mL penicillin, 100 mg/mL streptomycin, 2 mmol/L l‐glutamine, and 10 mmol/L Hepes and supplemented with 15% L929‐conditioned medium to generate bone marrow–derived macrophages (BMDMs). Medium was replaced every 3 days, and differentiated BMDMs were used for *in vitro* assays 7 days after isolation.

### 2.9 Statistical analysis

GraphPad Prism 8.4.2 was used for statistical analyses. To compare two experimental conditions, a two-tailed paired t test was used, and *p*<0.05 was considered statistically significant. For multiple comparisons, one-way ANOVA with Greenhouse–Geisser correction and Fisher’s LSD test or Sidak’s multiple comparisons test were applied. The minimal necessary amount of observations were used in each experiment to optimize the use of resources and, when applicable, minimize the use of animals.

### 2.10 Ethics

The approval of using human monocytes and monocytes-derived macrophages were granted by local ethical committee (regional ethics committee of Helse Sør-Øst; permit number S-05172). The conduction follows the ethical guidelines outlined in the World Medical Association’s Declaration of Helsinki for use of human tissue and subjects. The harvesting of mouse bone marrow cells was approved by the Norwegian National Animal Research Authority with FOTS project license numbers 22322 and 23000.

## 3 Results

### 3.1 Apigenin selectively inhibits IL-1β release from human monocytes and monocyte-derived macrophages

**Figure 1:**
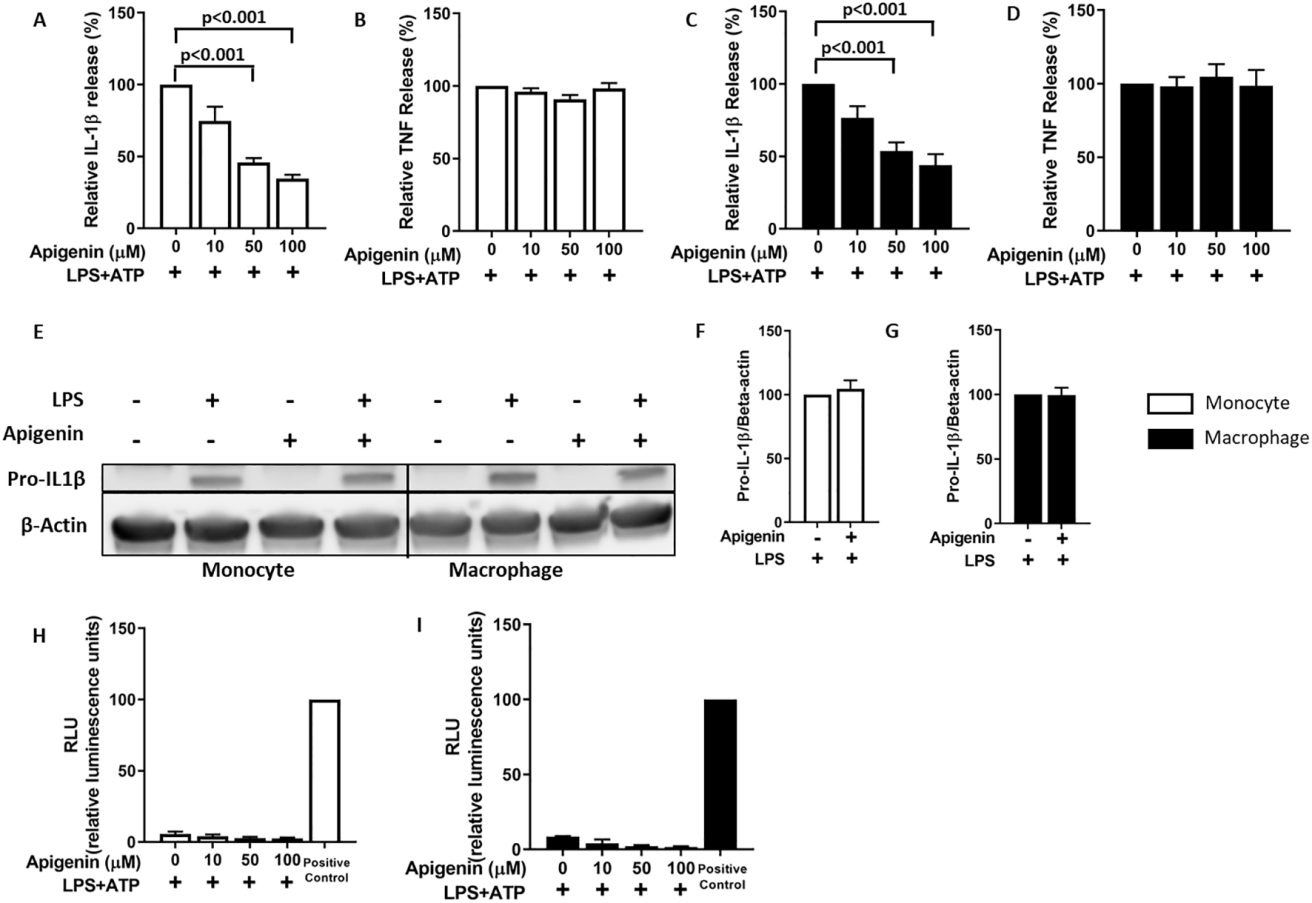
Apigenin specifically inhibits IL-1β release from human monocytes and monocyte derived macrophages. **(A–D)** Monocytes (n=4) or monocyte-derived macrophages (n=5) were incubated with 10 ng/mL LPS for 5 hours. Then cells were treated with or without apigenin at indicated doses for 30 min, followed by 30 min ATP (3 mM) activation. IL-1β and TNF were quantified in the conditioned media with ELISA. Columns are means with SEM. Data are normalized: cytokine release from control cells (treated with LPS+ATP only) are set as 100%. **(E–G)** Monocytes or monocyte-derived macrophages were first incubated with 10 ng/mL LPS for 5 hours followed by 30 min with or without 100 μM apigenin. Pro-IL-1β/β-actin ratios were quantified with Western blot analysis. Columns are mean with SEM (n=3). Data are normalized: pro-IL-1β β-actin ratios in control cells treated with LPS only are set as 100%. **(H, I)** Apigenin was evaluated for cytotoxicity with the ToxiLight assay in LPS-treated human monocytes and monocyte-derived macrophages. Positive controls are from lysed cells. Columns are mean with SEM (n=2). The indicated p-values were determined by one-way ANOVA with Greenhouse–Geisser correction and Fisher’s LSD test.

We hypothesized that apigenin could attenuate NLRP3 inflammasome activation through CD38 inhibition. However, apigenin may also inhibit NFκB activation (signal 1) in a CD38-independent manner (29). To circumvent this problem, we primed human primary monocytes and monocyte derived macrophages with LPS for 5 hours (signal 1) before incubating the cells with apigenin for only 30 minutes before activating NLRP3 inflammasomes with ATP (signal 2) for 30 minutes (**Figures 1A–G**). Apigenin effectively inhibited IL-1β release in a dose-dependent manner in both types of cells (**Figures 1B, C**) while TNF release (NFκB dependent) was not affected (**Figures 1B, D**). Furthermore, Western blot analysis showed that apigenin did not inhibit pro-IL-1β synthesis (**Figures 1E–G**) or featured any cytotoxic effect (**Figures 1H, I**).

### 3.2 Apigenin inhibits calcium flux in human monocyte derived macrophages

**Figure 2:**
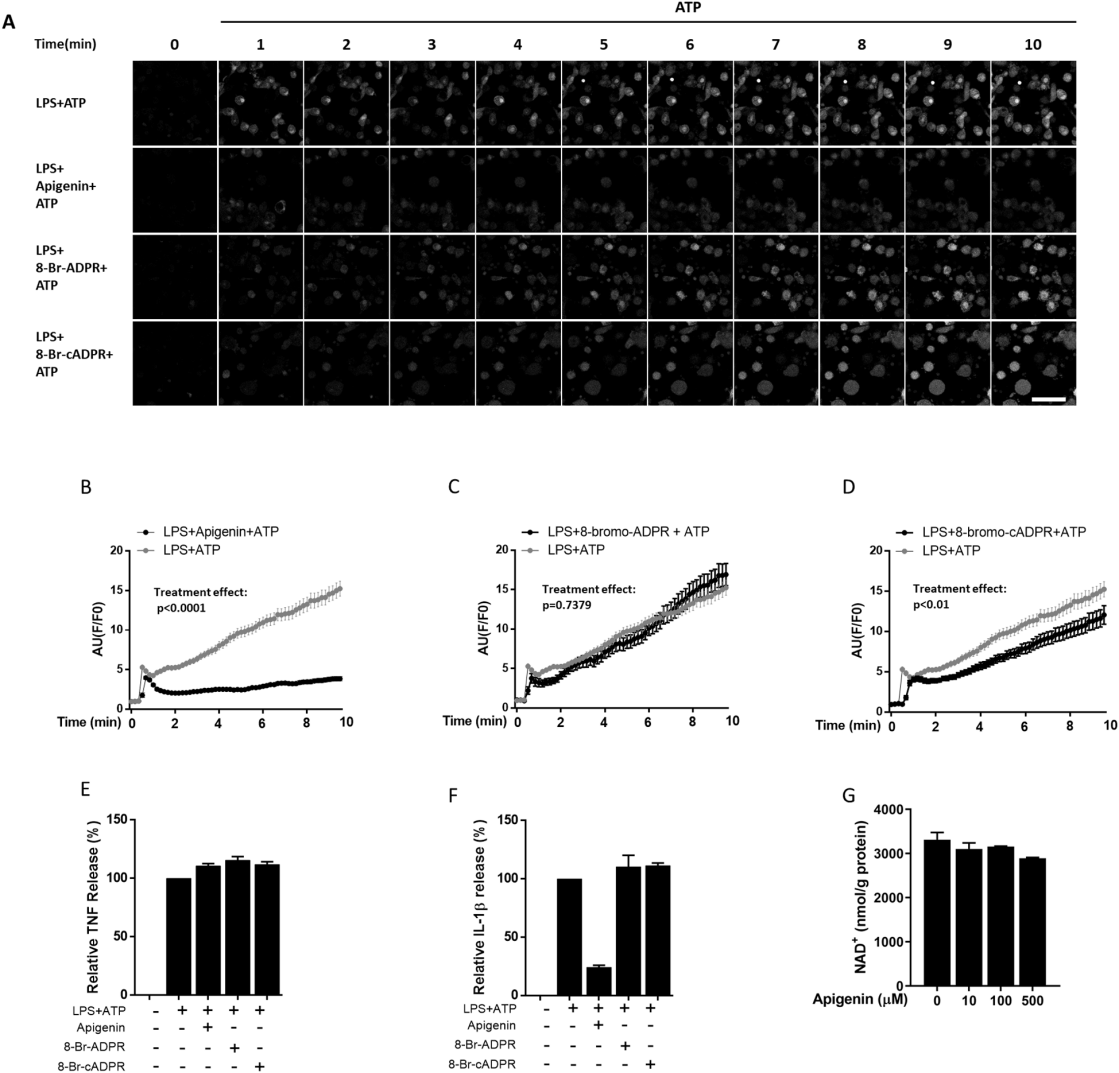
Apigenin attenuates calcium flux in human monocyte-derived macrophages. **(A)** Human monocyte derived macrophages (n=3) were incubated with 10 ng/mL LPS for 5 hours followed by 30 min with or without Apigenin (100 μM), 8-Br-ADPR (100 μM) or 8-Br-cADPR (100 μM). Cells were labeled with Fluor-4 AM for 5 min to show calcium mobilization and then activated with 3 mM ATP while immunofluorescence was quantified every 10 seconds for 10 minutes. A representative view from each condition in one biological repeat is shown. Scale bar (lower right) is 60 μm. **(B–D)** 20 cells were selected randomly from each condition and fluorescence was quantified every 10 seconds for 10 minutes to show calcium influx intensity as arbitrary units (AU). Treatment effects were tested with a two-way ANOVA test. **(E, F)** Human monocyte derived macrophages were incubated with 10 ng/mL LPS for 5 hours followed by 30 min with or without apigenin (100 μM), 8-Br-ADPR (100µM) or 8-Br-cADPR (100µM), then activated with 3 mM ATP for 30 min. IL-1β was quantified in the conditioned media with ELISA. Columns are mean with SEM (n=4). **(G)** Macrophages were incubated with apigenin for indicated doses for 30 minutes and cellular NAD-levels were quantified with bioluminescent assay.

It is well established that calcium influx is implicated in NLRP3 inflammasome activation (30). As CD38 can convert NAD^+^ into the Ca^2+^ mobilizing second messengers ADPR and cADPR, a role for CD38 in this NLRP3 inflammasome activating process has been suggested (31, 32). To investigate whether apigenin may inhibit IL-1β release by attenuating Ca^2+^ flux to the cytosol, we performed calcium live imaging on human monocyte derived macrophage. Cells were primed with LPS for 5 hours and incubated with apigenin or the second messenger antagonists 8-Bromo-ADPR or 8-Bromo-cADPR, respectively, before activation with ATP (**Figures 2A–D**). Apigenin effectively attenuated calcium influx after ATP activation (**Figure 2B**). In contrast, no effect of 8-Bromo-ADPR could be observed while 8-Bromo-cADPR moderately attenuated Ca^2+^ flux (**Figures 2C, D**). Again, apigenin inhibited IL-1β release without interfering with TNF release. However, neither 8-Bromo-ADPR nor 8-Bromo-cADPR (which moderately attenuated apigenin-induced Ca^2+^ flux) had any significant effect on IL-1β or TNF release (**Figures 2E, F**). Finally, since CD38 is a major consumer of cellular NAD^+^, we also examined whether apigenin at increasing doses for 30 minutes could alter cellular NAD^+^ level in human primary macrophages. However, no changes in cellular NAD^+^ were observed (**Figure 2G**).

### 3.3 The selective CD38 inhibitor 78c attenuates NLRP3-dependent IL-1β release

**Figure 3:**
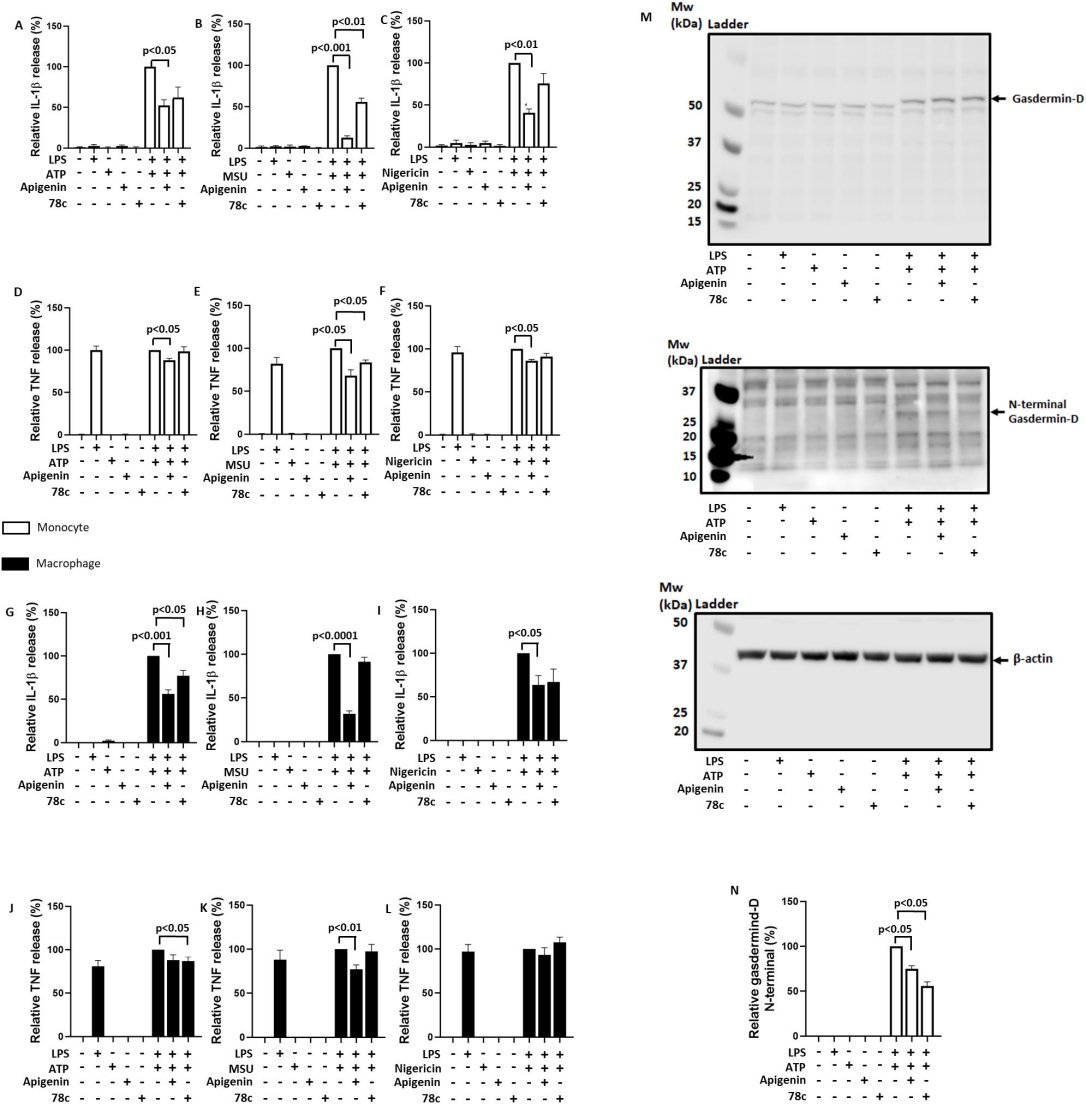
Comparative inhibition of IL-1β and TNF release with apigenin and 78c. Monocytes (n=3) or monocyte-derived macrophages (n=6) were incubated with 10 ng/mL LPS for 5 hours (3.5 hours for MSU) followed by 30 min with or without Apigenin (100 μM) or 78c (10μM). Cells were activated with 3 mM ATP for 30 minutes **(A, D, G, J)** or 15 minutes **(M, N**), 200 μg/mL MSU for 2 hours **(B, E, H, K)** or 10 μM nigericin for 30 minutes **(C, F, I, L)**. IL-1β and TNF were quantified in conditioned media with ELISA. Gasdermin-D, N-terminal gasdermin-D and β-actin were examined by western blot. N-terminal gasdermin-D/β-actin ratio were quantified and normalized to 100% in positive control cells (LPS primed cells activated with ATP). Columns are mean with SEM. The indicated p-values were determined by one-way ANOVA with Greenhouse–Geisser correction and Fisher’s LSD test.

Apigenin inhibits CD38 signaling but has also been proposed to inhibit the extracellular ATP receptor P2X7R (33). To clarify whether CD38 inhibition is the main mechanism for attenuating IL-1β release by apigenin, we treated LPS (signal 1) primed human monocytes and human monocyte- derived macrophages with the alleged specific CD38 inhibitor 78c (34) for 30 minutes prior to signal 2 and the effect was compared to that of apigenin. Furthermore, to investigate if CD38 has a general implication in NLRP3 activation, the cells were activated with either ATP, MSU or nigericin. 78c significantly reduced IL-1β release induced by MSU in monocytes as well as by ATP in macrophages (**Figures 3B, G**). Apigenin, however, was comparably more efficient and significantly reduced IL-1β release induced by ATP and MSU in both monocytes and macrophages (**Figures 3A, B, G–I**). Intriguingly, however, only apigenin significantly attenuated nigericin-induced IL-1β release (**Figures 3C, I**). Moreover, in this experiment apigenin also reduced TNF release from monocytes treated with ATP, and monocytes and macrophages treated with MSU (**Figures 3D–F, J**). Finally, both 78c and apigenin attenuated the ATP-induced formation of N-terminal gasdermin-D in LPS-primed monocytes (**Figures 3M, N**), another endpoint for NLRP3 inflammasome activation. Hence, these data support that CD38 is involved in NLRP3 inflammasome activation in human monocytes and monocyte-derived macrophages, but also show a broader inhibitory effects of apigenin.

#### 3.3.1 The ATP hydrolase apyrase inhibits NLRP3 activation by ATP and MSU, but not nigericin

**Figure 4:**
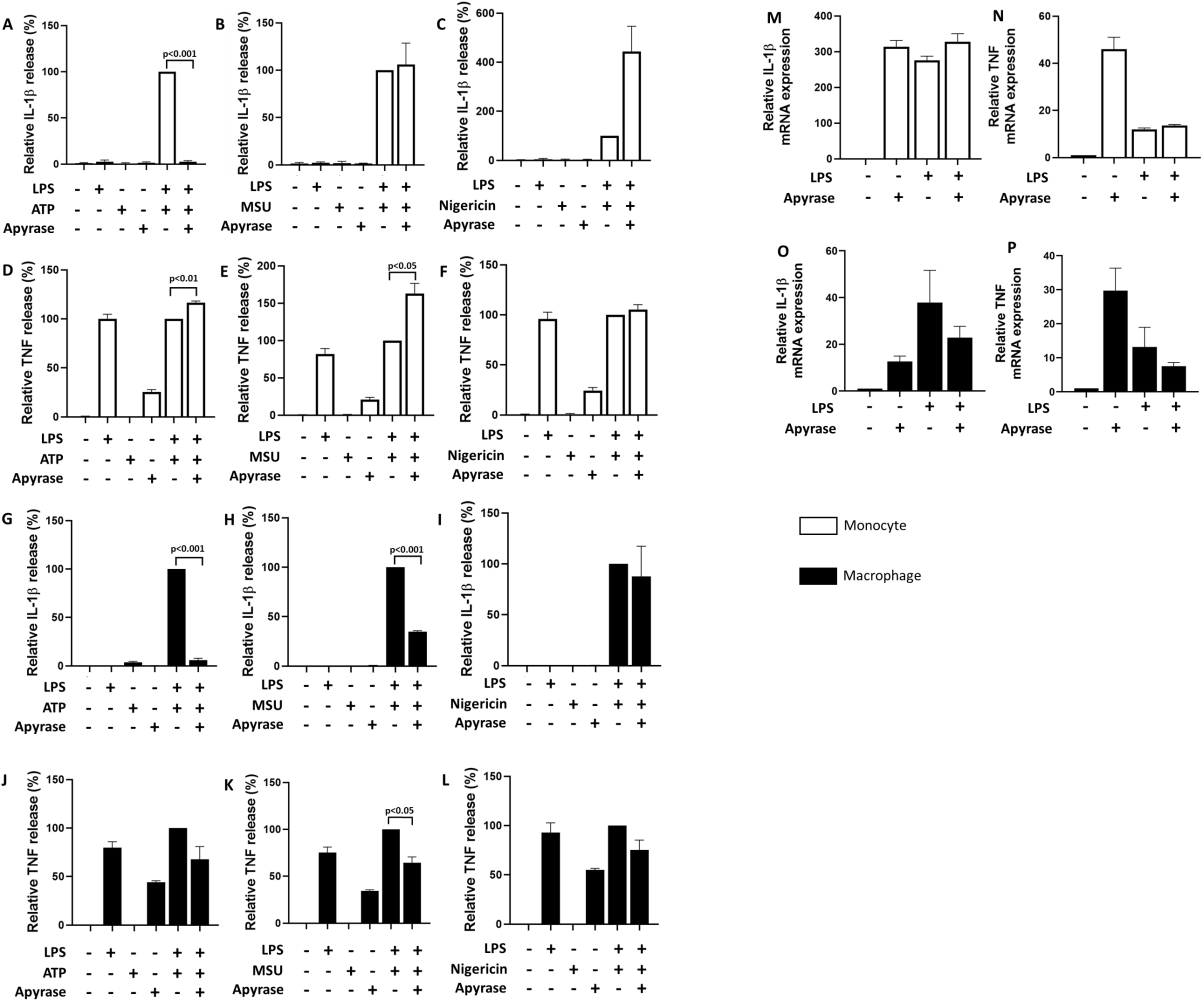
Inhibition of IL-1β and TNF release with apyrase. Monocytes or monocyte-derived macrophages (n=3) were incubated with 10 ng/mL LPS for 5 hours (3.5 hours for MSU) followed by 30 min with or without apyrase (10U/mL). Cells were activated with 3 mM ATP for 30 minutes **(A, D, G, J)**, 200 μg/mL MSU for 2 hours **(B, E, H, K)** or 10 μM nigericin for 30 minutes **(C, F, I, L)**. IL-1β and TNF were quantified in conditioned media with ELISA. Columns are mean with SEM. ELISA data are normalized: cytokine release from positive control cells (treated with LPS+ATP only) are set as 100%. Apyrase were tested for pro-inflammatory features separately **(M–P)** by quantifying IL-1β and TNF mRNA by qPCR. The indicated p-values were determined by one-way ANOVA with Greenhouse–Geisser correction and Fisher’s LSD test. qPCR data are normalized: mRNA level in untreated cells are set as 1.

Since apigenin seemed comparatively more efficient than the specific CD38 inhibitor 78c in attenuating NLRP3-dependent IL-1β release, we suspected that apigenin may also inhibit NLRP3 through CD38-independent mechanisms. Polyphenols in general have previously been proposed to inhibit the interaction of extracellular ATP with P2X7R (33). However, apigenin also attenuated IL-1β release induced by MSU and nigericin (**Figures 3B, C, H, I**). To clarify whether apigenin may interact directly with P2X7R, LPS-primed human monocytes and monocyte derived macrophages were incubated with apyrase 30 minutes prior to NLRP3 activation with ATP, MSU or nigericin (**Figure 4**). As expected, apyrase strongly attenuated IL-1β secretion induced by ATP in both cell types (**Figures 4A, G**). Furthermore, apyrase also significantly and efficiently attenuated IL-1β release from MSU activated macrophages (**Figure 4H**). Apyrase did not reduce IL-1β secretion induced by nigericin (**Figures 4C, I**). Thus, our data do not support the involvement of P2X7R in nigericin-mediated NLRP3 activation. Altogether, our data support that apigenin attenuates NLRP3 activation independently of P2X7R interaction. Finally, apyrase alone also induced IL-1β and TNF transcription (**Figures 4M, N**), as well as TNF secretion (**Figures 4D–F, J–L**), and even enhanced TNF release in ATP or MSU activated monocytes, suggesting it was contaminated with pathogen associated molecular patterns (PAMPs) which activated signal 1 through Toll-like receptors.

### 3.4 The NLRP3 inflammasome is fully functional in CD38^-/-^ macrophages

**Figure 5:**
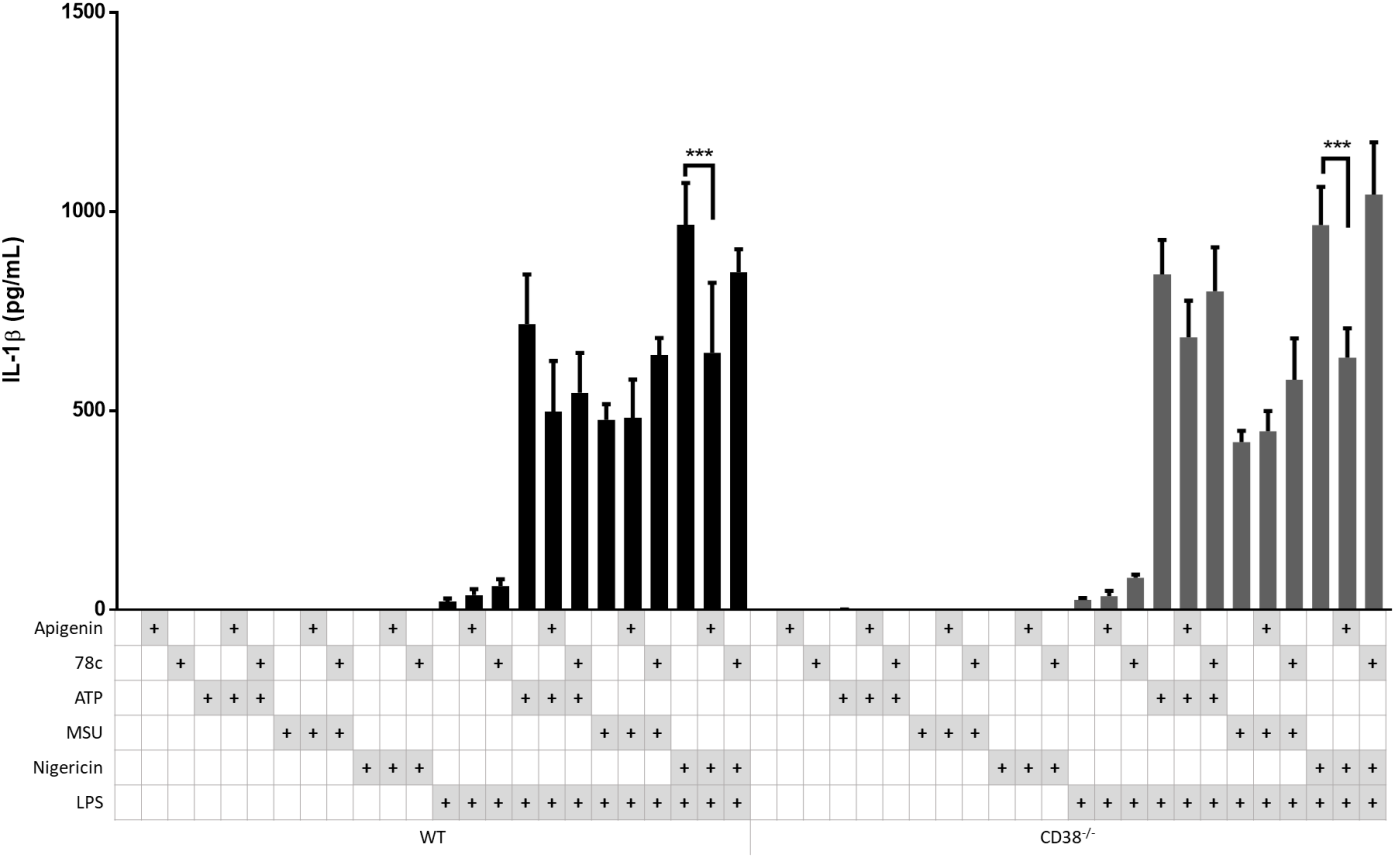
Apigenin inhibits IL-1β in both WT and CD38^-/-^ macrophages. Bone marrow-derived macrophages (BMDM) were isolated from CD38^-/-^ mice and WT littermates (n=3). The cells were incubated with 10 ng/mL LPS for 5 hours with or without Apigenin (100 μM) or 78c (10μM), then activated with either 3 mM ATP for 30 minutes, 200 μg/mL MSU for 2 hours or 10 μM nigericin for 30 minutes. IL-1β were quantified in conditioned media with ELISA. Columns are mean with SEM. Based on the column pattern, tentative meaningful differences were tested for statistical significance (i.e., LPS+ ATP vs LPS + ATP with Apigenin, and LPS + Nigericin vs LPS + Nigericin with Apigenin. The indicated p-values were determined with Sidak’s multiple comparisons test.

Although both apigenin and 78c could attenuate NLRP3-dependent IL-1β release, a stronger experiment for testing the relevance of CD38 is activating NLRP3 inflammasomes in CD38^-/-^ bone marrow derived mouse macrophages in comparison with corresponding wild type (WT) cells. Hence, CD38^-/-^ and WT BMDM were incubated with medium only or LPS for 5 hours and inflammasomes activated with ATP, MSU or nigericin. The effects of apigenin and 78c were also tested (**Figure 5**). Surprisingly, the NLRP3 inflammasome mediated IL-1β activation was fully functional in CD38^-/-^ macrophages compared to WT macrophages. Furthermore, apigenin significantly attenuated nigericin-induced IL-1β release with a similar magnitude in both genotypes. 78c had no significant effect on either genotype. These results strongly suggest that CD38 has no role in NLRP3 inflammasome activation, at least in mice, and that the effects of apigenin in the human experiments are not related to CD38.

## 4 Discussion

**Figure 6:**
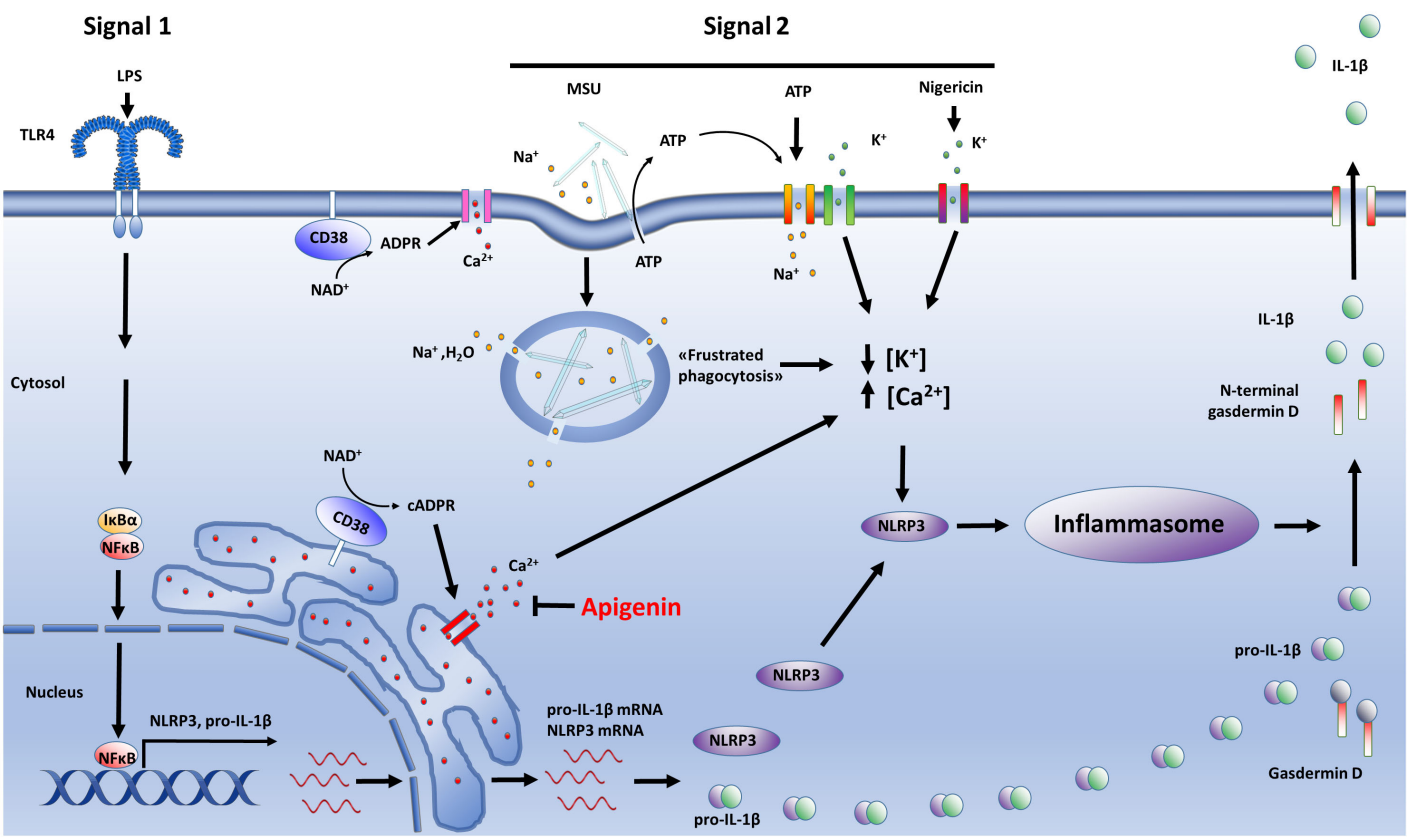
Summary. The activity of NLRP3 inflammasomes are tightly regulated through two independent steps: Signal 1 activates the transcription factor NFκB which induces NLRP3 and pro-IL-1β expression (left side). Signal 2, which can be one of several danger signals, including MSU, ATP or nigericin, activates NLRP3 through a major change in cytosolic potassium and calcium levels (middle). This results in the assembly of inflammasome complexes in which caspase-1 is activated. Inflammasome-contained caspase-1, in turn, cleaves and activates pro-IL-1β and gasdermin D (right side). N-terminal gasdermin D makes pores in the plasma membrane, releasing active IL-1β and other molecules. We hypothesized that CD38 contributes to NLRP3 activation through its role in calcium release (left side), but our data do not support this. Apigenin inhibits ATP-induced Ca^2+^ influx and attenuate NLRP3 activation by all the tested activators (ATP, MSU, nigericin), albeit independent of CD38.

In this study we hypothesized that CD38 is implicated in NLRP3 activation in human monocytes and macrophages. Our hypothesis was initially supported by the following findings: i) Apigenin, a well-characterized inhibitor of CD38 activity, effectively attenuated ATP-induced IL-1β release in a dose-dependent manner in LPS-primed cells without interfering with TNF secretion nor pro-IL-1β protein levels; ii) Apigenin effectively inhibited ATP-mediated Ca^2+^ flux in LPS-primed monocyte-derived macrophages; iii) Both apigenin and 78c, a specific CD38 inhibitor, attenuated NLRP3-dependent IL-1β release and N-terminal gasdermin-D formation. However, we also showed that NLRP3 inflammasomes are still fully functional in CD38^-/-^ macrophages. Hence, our data show that CD38 has no important role in NLRP3 inflammasome induction or activation, nor does apigenin inhibit NLRP3 inflammasomes through CD38. However, we cannot rule out the possibility that different mechanisms related to CD38 function operate in mice and humans (35–37). The interpretation of our findings are summarized in **Figure 6**.

CD38 is crucial for innate immune responses; CD38 knockout mice succumb early to *L. monocytogenes* and *S. pneumoniae* infection (16, 17). Furthermore, CD38 expression can be induced by TNF (38) and is highly expressed by immune cells such as monocytes and M1 macrophages (14, 15). Until quite recently, the implication of CD38 in NLRP3 activation had not been elucidated. In 2018, Yan et al. reported that coronary artery smooth muscle cells obtained from CD38 knockout mice featured increased NLRP3 activity compared to corresponding WT cells. The authors attributed this to disturbed autophagy in the CD38^-/-^ cells (18). However, more recently, Li et al. reported that CD38 gene silencing with siRNA attenuated Ca^2+^ flux and NLRP3 activation in mouse vascular smooth muscle cells exposed to high glucose, suggesting that CD38 may contribute in the pathogenesis of diabetic vascular disease (19). This discrepancy may be caused by the permanent and total CD38 gene loss in the former experimental model as opposed to the temporary and non-complete CD38 inhibition with siRNA by Li et al. and by pharmacological CD38 inhibitors in our experiments. Notwithstanding, our present findings do not support the conclusion of Li et al. that CD38 promotes NLRP3 activation. Importantly, however, it has been shown that NLRP3 activation may differ between different cell types (39), and this could potentially also include differences between macrophages and smooth muscle cells. In our experiments, NLRP3 activation in monocytes and macrophages, included CD38^-/-^ BMDMs, was tested with three different danger signals: extracellular ATP, urate crystals and nigericin. These danger signals activate NLRP3 by different but converging cellular events, as indicated in **Figure 6**, and are regarded as the most important NLRP3 activators (40). However, we cannot exclude that CD38 may have a role in NLRP3 activation mediated by other danger signals or pathogen associated molecular patterns than the once tested in our experiments. Another potential limitation in our experiment is the low number of observations (n=3) of WT and CD38^-/-^ BMDMs. While small differences could theoretically emerge with a larger dataset, this seems unlikely, as our data demonstrate nearly identical patterns of apigenin’s effect on IL-1β release in both WT and CD38^-/-^ BMDMs.

78c is a thiazoloquinazolinone derivative found to have potent inhibitory activity against CD38’s cyclase/hydrolase function (34). The claim of specificity is based on 78c’s failure to inhibit other NAD^+^ metabolizing enzymes such as poly [ADP-ribose] polymerase 1 (PARP1), sirtuin 1 and nicotinamide phosphoribosyltransferase (NAMPT) (41). However, whether 78c targets other proteins unrelated to NAD^+^ metabolism is not known. Thus, 78c could inhibit the NLRP3 inflammasomes independent of CD38, as our data suggest.

Unlike 78c, apigenin is not an alleged specific CD38 inhibitor, and its anti-inflammatory mechanisms are not clear. As a flavonoid, apigenin could act as a free-radical scavenger and an antioxidant, thus affecting several cellular processes. However, compared to other flavonoids, apigenin seems to be a rather poor direct scavenger of free radicals and reactive oxidative species (ROS) (42–44). Although apigenin has been suggested to attenuate NADPH oxidases (45), others have failed to replicate this finding (44). Notwithstanding, apigenin seems to have significant anti-inflammatory effects *in vivo* (46, 47), which could in part be explained by inhibited NFκB activation (29). In our study, however, the cells were already primed with LPS prior to short term (30 minutes) apigenin exposure, and apigenin could inhibit NLRP3 (signal 2) without affecting NFκB activation (signal 1), as shown by unaffected TNF secretion (**Figures 1**, **2**). However, a small but significant apigenin-induced decrease in TNF release was occasionally observed (**Figure 3**), suggesting an attenuating effect on signal 1. As expected, this was most prominent in cells activated with MSU crystals, in which case the total apigenin exposure time lasted 2.5 hours and is in line with previous findings (29). Moreover, apigenin has been proposed, along with other flavonoids, to inhibit NLRP3 activation through interaction with P2X7R (25). P2X7R is expressed in most tissues and is essential for NLRP3 activation by extracellular ATP. When ligated by ATP, P2X7R promotes Ca^2+^ and Na^+^ influx across the plasma membrane while the K^+^ channel Two-pore domain Weak Inwardly rectifying K^+^ channel 2 (TWIK2) is activated and mediates K^+^ efflux (**Figure 6**). The resulting reduced cytosolic K^+^ level is thought to be a common denominator shared by most, perhaps all, NLRP3 activating pathways (8). MSU crystals and other NLRP3 activating particulate matters have been proposed to reduce cytosolic K^+^ concentration by inducing leakage of lysosomal content and overload the cytosol with Na^+^ and water in a process termed “frustrated phagocytosis” (48). In contrast, the pore-forming toxin nigericin seem to mediate K^+^ efflux directly (49). In our experiments, apigenin appeared to be the more potent NLRP3 inhibitor as compared to the allegedly specific 78c. Even so, considering that CD38 is most likely not involved in NLRP3 activation after all, the dampening effect of 78c is most likely due to an unknown off-target effect, perhaps similar to that of apigenin.

Since apigenin effectively inhibited NLRP3 activation by ATP, MSU and nigericin, we investigated the role of P2X7R for each of these NLRP3 activators by hydrolyzing extracellular ATP with apyrase. Indeed, apyrase diminished IL-1β release induced by ATP or MSU. However, as opposed to apigenin, apyrase could not hinder nigericin-induced IL-1β release. Hence, our data support that apigenin attenuates NLRP3 inflammasome formations independently of P2X7R.

Apigenin strongly inhibited Ca^2+^ flux to the cytosol, while the cADPR antagonist was less effective and the ADPR antagonist showed no effect. Hence, our data suggest that apigenin may reduce NLRP3 activity by attenuating the Ca^2+^ flux, albeit without involving CD38.

In 2012, Riteau et al. reported that MSU, silica and alum crystals induced ATP secretion from PMA-primed THP-1 cells, and that a pharmacological inhibitor of P2X7R attenuated IL-1β release induced by silica or alum crystals. However, the authors could not observe attenuated IL-1β release from bone marrow-derived macrophages obtained from P2X7R knockout mice compared to corresponding WT cells, which they suggest may be caused by redundant compensatory mechanisms in the P2X7R^-/-^ cells (50). In this study we observed that apyrase served as a potent inhibitor of MSU-induced IL-1β release from monocyte derived macrophages, further supporting a role for the ATP/P2X7R signal 2 pathway in NLRP3 activation by particulate matters. Why we did not observe this effect of apyrase in monocytes is a puzzling question, in particular since apyrase effectively inhibited the effect of high extracellular ATP levels (3 mM). Apyrase was clearly contaminated with microbial molecular patterns, as seen by TNF secretion induced by apyrase alone (**Figure 4**), which may have interfered with our monocyte experiment. It is also possible that particulate matters activate monocytes differently from macrophages.

Riteau’s idea of a redundant compensatory mechanisms in the P2X7R^-/-^ cells could, of course, also apply to our CD38^-/-^ BMDM. Nonetheless, apigenin still reduced IL-1β release significantly from CD38^-/-^ and WT macrophages treated with LPS and nigericin. Thus, we do not find a major role for CD38 in direct NLRP3 inflammasome activation, nor does the inflammasome inhibiting effect of apigenin seem to be mediated through CD38. However, further studies in this area of research should also include *in vivo*/*ex vivo* studies in humans as well as studies in bone marrow transplanted CD38^-/-^ mice.

In summary, our data support that apigenin attenuates NLRP3 inflammasome activation through CD38-independent inhibition. Furthermore, our data do not support a role for CD38 in NLRP3 inflammasome activation in monocyte/macrophages. This study further supports the potential of apigenin as an anti-inflammatory drug.

## Data Availability

The raw data supporting the conclusions of this article will be made available by the authors, without undue reservation.
