# Supplementary material for: Apigenin inhibits NLRP3 inflammasome activation in monocytes and macrophages independently of CD38
Source: Front Immunol. 2025 Jan 7;15:1497984. doi: 10.3389/fimmu.2024.1497984 (PMC11746122; doi:10.3389/fimmu.2024.1497984)
Supplement: Supplementary file 1 [file Table1.docx]

| **Supplemental table 1: Primer sequences used for genotyping** | |
| --- | --- |
| **Target** | **Sequence (5’ 3’)** |
| Mutant forward primer | AATCCATCTTGTTCAATGGCCGATC |
| Wild type forward primer | CACCATAAGAGGGGAAGCAA |
| Reverse primer | TGCCAAAAGTGCAGAAGAGA |
