# Supplementary material for: Apigenin inhibits NLRP3 inflammasome activation in monocytes and macrophages independently of CD38
Source: Front Immunol. 2025 Jan 7;15:1497984. doi: 10.3389/fimmu.2024.1497984 (PMC11746122; doi:10.3389/fimmu.2024.1497984)
Supplement: Supplementary file 2 [file DataSheet1.pdf]

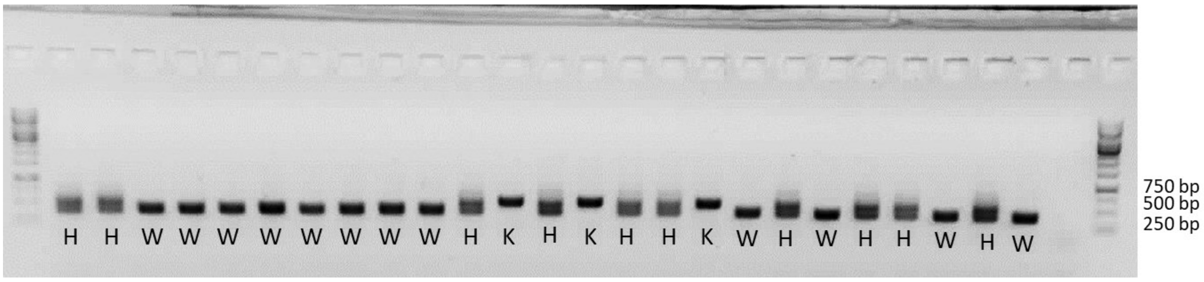

Supplementary figure 1. Genotyping of WT (W), heterozygous CD38$^{+/-}$ (H) and CD38$^{-/-}$ (K) mouse littermates. Band sizes for the WT CD38 genotype is 417 base pairs (bp), while the CD38$^{-/-}$ band size is 600 bp, in accordance with the specifications provided from the Jackson laboratory (https://www.jax.org/strain/003727#). The indicated CD38$^{-/-}$ mice were sacrificed for the experiment presented in figure 5.
