# Supplementary material for: Apigenin inhibits NLRP3 inflammasome activation in monocytes and macrophages independently of CD38
Source: Front Immunol. 2025 Jan 7;15:1497984. doi: 10.3389/fimmu.2024.1497984 (PMC11746122; doi:10.3389/fimmu.2024.1497984)
Supplement: Supplementary file 3 [file DataSheet2.pdf]

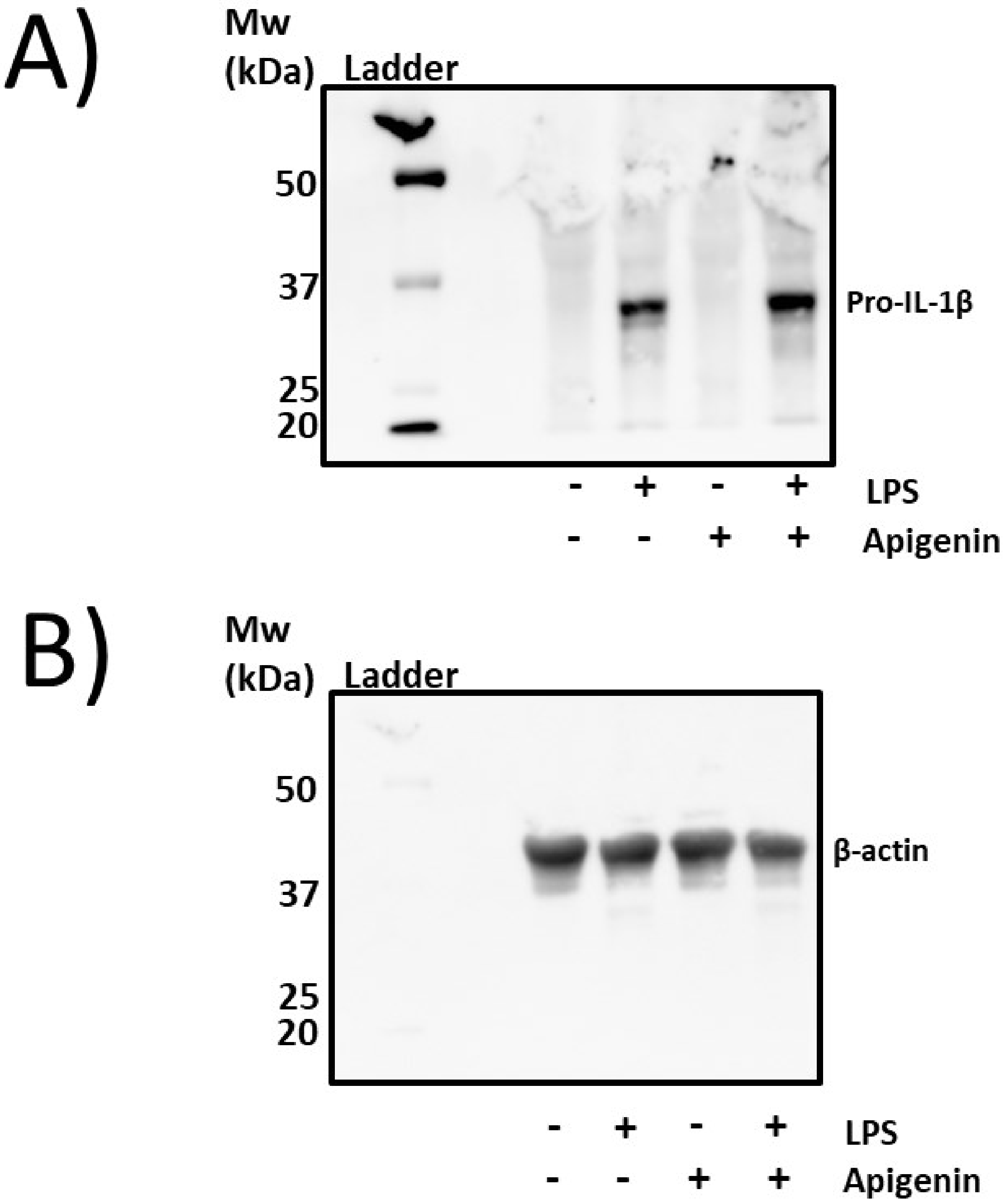

A) Pro-IL-1β size shown with molecular ladder from monocytes treated with 10 ng/mL LPS for 5 hours with or without apigenin (100 μM) for 30 minutes. **B)** Corresponding β-actin.
